# Gallbladder Volvulus: An Uncommon Twist in Biliary Pathology

**DOI:** 10.7759/cureus.20469

**Published:** 2021-12-16

**Authors:** Xinlin Chin, Jessica Y Ng

**Affiliations:** 1 Surgery, Sunshine Coast University Hospital, Birtinya, AUS; 2 School of Medicine, Griffith University, Birtinya, AUS; 3 Surgery, Princess Alexandra Hospital, Woolloongabba, AUS; 4 School of Medicine, Griffith University, Southport, AUS

**Keywords:** laparoscopic cholecystectomy, hepatocystic ligament, cystic duct, cholecystitis, gall bladder diseases, gallbladder torsion, gallbladder volvulus

## Abstract

Gallbladder volvulus (GBV) is an extremely rare disease, which presents similarly to acute cholecystitis. It has an incidence of less than 0.1% among urgent cholecystectomies and one in 356,000 hospital admissions. We report the case of a 92-year-old female with a three-day history of abdominal pain that had acutely worsened and localized to the right upper quadrant over the past 24 hours. Physical examination revealed a tender palpable mass in the right upper quadrant. Laboratory investigations demonstrated elevation of the white cell count and liver enzymes while CT abdomen showed a thick-walled gallbladder with an abrupt cut-off of the cystic duct suggestive of gallbladder volvulus. A laparoscopic cholecystectomy revealed a massively distended gangrenous gallbladder which has volved on the hepatocystic ligament. We present this case to demonstrate the radiological and intraoperative findings of GBV and to highlight the importance of early intervention to avoid life-threatening complications.

## Introduction

Gallbladder volvulus (GBV) is an extremely rare disease, which presents similarly to acute cholecystitis [1-4]. It has an incidence of less than 0.1% among urgent cholecystectomies and one in 356,000 hospital admissions [4,5]. Literature review showed approximately 500 reported cases since its first description in 1898 [1,6]. While GBV occurs more frequently among the elderly (age 60 to 80) with a female predominance of 3.5:1, less than one-sixth of the cases have been reported among the paediatric group with a male predominance of 2.5:1 [6,7]. Loss of supportive visceral fat and liver atrophy among the elderly along with the presence of a long mesentery may lead to GBV [3,8]. The mortality rate is 100% without treatment and approximately 6% with a prompt intervention [2].

## Case presentation

A 92-year-old female presented to the emergency department with a three-day history of abdominal pain which had acutely worsened and localized to the right upper quadrant (RUQ) over the past 24 hours. Her past medical history was significant for ischaemic heart disease, paroxysmal atrial fibrillation, and chronic kidney disease. She had a past surgical history significant for transcatheter aortic valve implantation and pacemaker insertion. On examination, she was afebrile with a pulse rate of 65 beats per minute. Blood pressure and respiratory rate were normal. Abdominal examination revealed a soft, distended abdomen with a tender palpable mass in the RUQ. A positive Murphy’s sign was noted.

**Figure 1:**
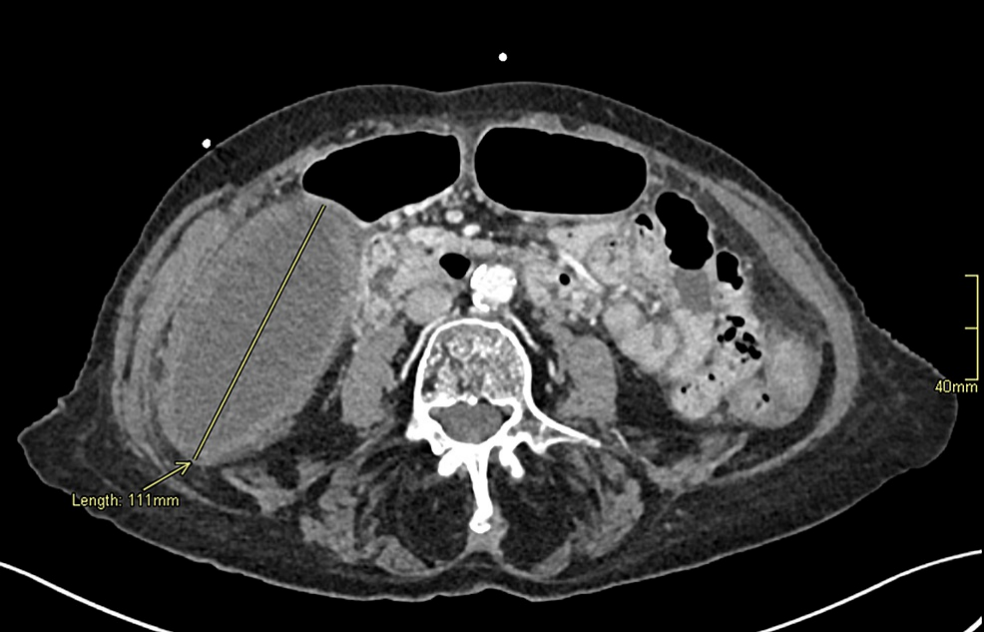
Axial contrast-enhanced computed tomography of the abdomen and pelvis Yellow arrow and line show a thick-walled, distended gallbladder measuring 11.1 cm in the right abdominal cavity

**Figure 2:**
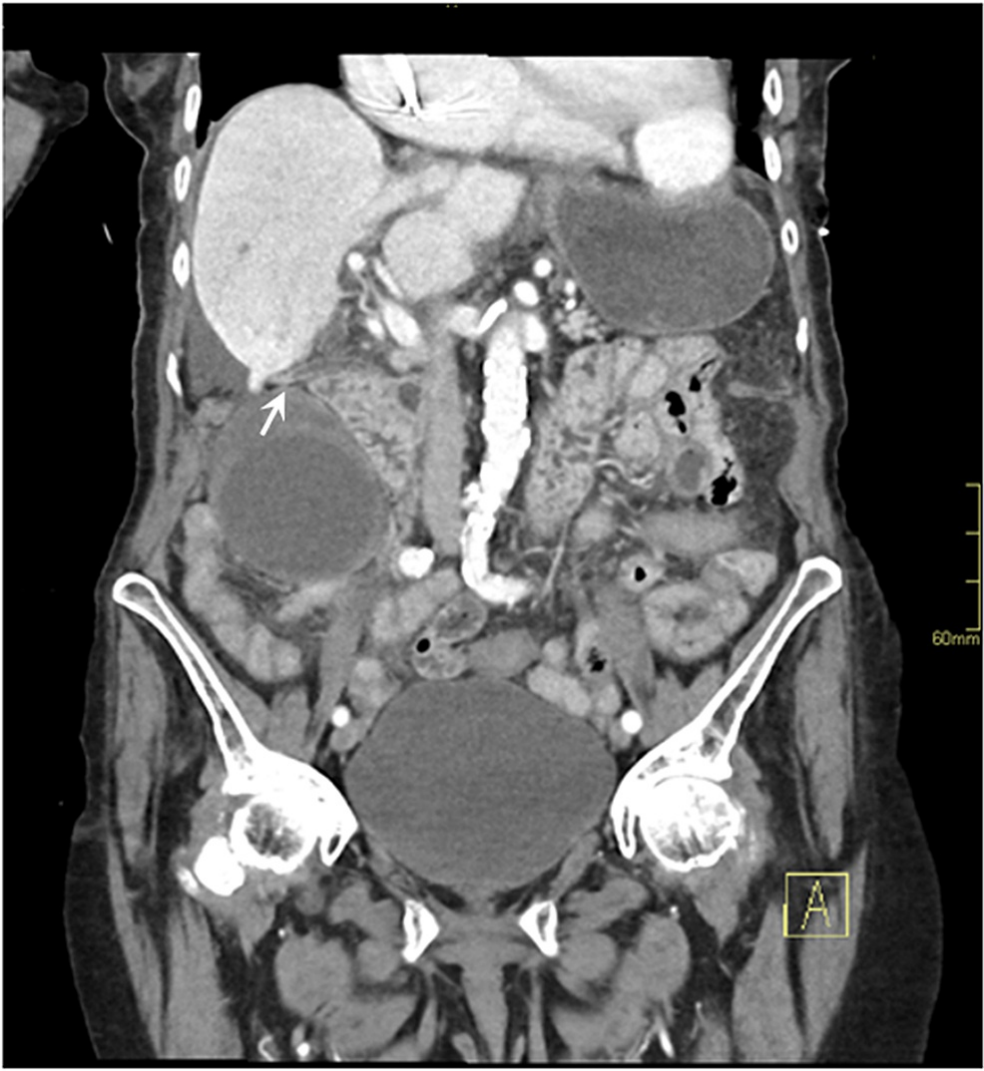
Coronal contrast-enhanced computed tomography of the abdomen and pelvis The white arrow shows abrupt cut-off of the cystic duct suggestive of gallbladder volvulus

Laboratory investigations showed an elevated white cell count of 21.4 x 10^9^/L and a neutrophil count of 19.14 x 10^9^/L. Liver function test revealed elevation of the alanine transaminase (59 unit/L) and aspartate transaminase (58 unit/L). Alkaline phosphatase, gamma-glutamyl transferase, and bilirubin within normal limits. Renal function test showed an estimated glomerular function rate of 37 mL/min/1.73 m^2^ and creatinine of 112 µmol/L. Computed tomography imaging of the abdomen and pelvis (CTAP) showed a thick-walled gallbladder measuring 11.1 cm with an abrupt cut-off of the cystic duct suggestive of gallbladder volvulus around the cystic duct (Figures 1, 2).

**Figure 3:**
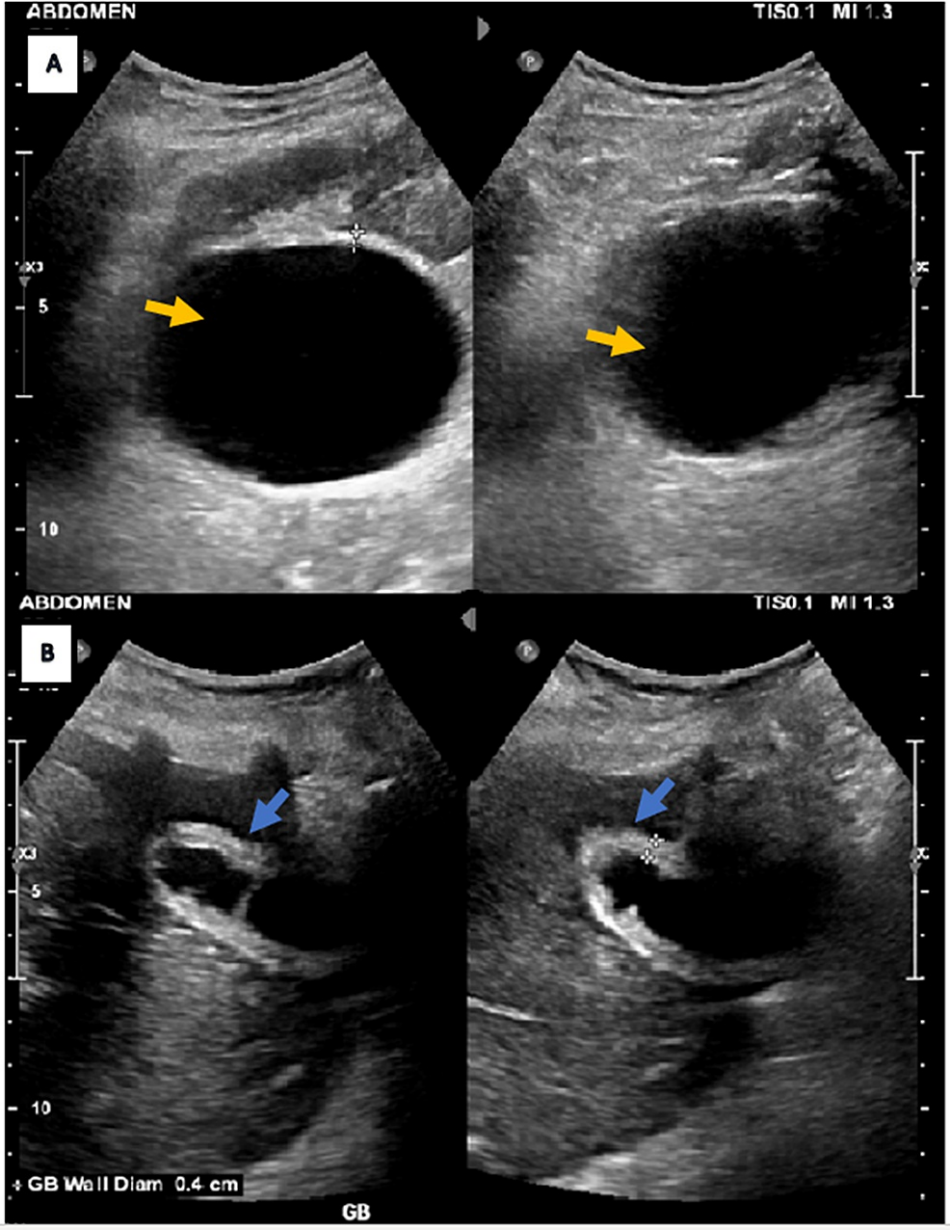
Ultrasound abdomen (A) Yellow arrows show a grossly distended gallbladder (B) Blue arrows show thickened wall measuring 4 mm

There was mild dilatation of the intra- and extrahepatic biliary duct system and widespread free fluid in the abdominal cavity. Ultrasound abdomen demonstrated a grossly distended gallbladder with a thickened wall (4 mm), pericholecystic fluid, and a positive sonographic Murphy’s sign consistent with acute inflammatory cholecystitis (Figure 3). The patient was booked for laparoscopic cholecystectomy. 

**Figure 4:**
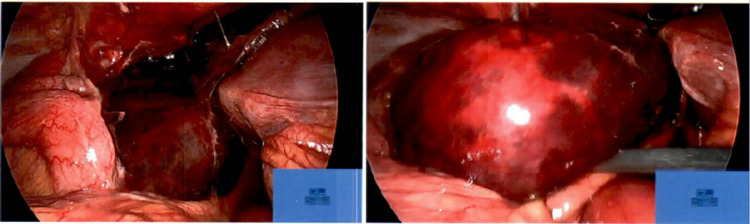
Intraoperative findings of the laparoscopic cholecystectomy showing a distended and gangrenous gallbladder The figure in the right-hand side panel shows a closer view of the distended and gangrenous gallbladder

Intraoperatively, a massively distended (length: 120 mm, fundal diameter: 55 mm) gangrenous gallbladder was noted to have volved on the hepatocystic ligament (Figure 4). The gallbladder was decompressed, de-rotated on mesentery back to restore the anatomical position, and removed. Histopathology report described an acute suppurative, necrotising, and haemorrhagic cholecystitis.

## Discussion

GBV can be complete (more than 180°) or incomplete (less than 180°) based on the degree of rotation around the cystic duct, and clockwise (gastric peristalsis) or anticlockwise (colonic peristalsis) depending on the type of peristalsis involved [1,2,9]. While the main etiology of GBV is unknown, possible mechanisms include a lack of adherence to the liver (floating gallbladder) and a tendency to twist around the cystic duct [1]. The four anatomic variants that predispose to the formation of GBV include congenital absence of the gallbladder mesentery, elongation, and increased motility of the gallbladder mesentery due to aging, a detached gallbladder fundus from the liver, and a fixed normal gallbladder to a mobile hepatic lobe [2]. These increase the risk of the freely suspended gallbladder twisting along the axis of the cystic duct and artery leading to GBV [2,4]. Obstruction of the cystic duct and strangulation of the cystic artery in GBV causes gallbladder ischaemia which results in massive distension and gangrene of the gallbladder wall [8].

GBV and cholecystitis share common presentation features like RUQ tenderness, nausea, and vomiting but GBV has a higher risk of perforation and necrosis [2,10]. The clinical features of GBV are grouped into a “triad of triads” which include patient characteristics (thin, elderly, chronic chest disease or kyphotic); symptoms (typical abdominal pain, early onset of vomiting, nausea); and physical signs (palpable abdominal mass, lack of toxaemia or jaundice, pulse-temperature discrepancy) [3,10]. GBV should be considered as a differential diagnosis among patients fulfilling most of the features [11]. Five of the nine features were present in our patient.

Paediatric patients with GBV may present with an acute on chronic RUQ tenderness, pain more severe than that of acute appendicitis, gradual onset of fever and a RUQ mass which is palpable under general anaesthesia [12]. Apart from acute cholecystitis, differential diagnoses that should be considered among the paediatric population include acute appendicitis, congenital dilatation of the bile duct, choledocholithiasis, intussusception, and intestinal volvulus [13].

Abdominal ultrasound and CTAP are the primary preoperative imaging modalities described in the literature [2]. Potential radiological features to diagnose GBV preoperatively include fluid collection demonstrating a horizontally positioned floating gallbladder, a well-enhanced cystic duct on the right of the gallbladder, and signs of ischaemia or inflammation like hyperattenuation and thickening of the gallbladder wall [1,3,6]. Angulation of the gallbladder neck (“beak sign”), twisting of the vascular pedicle (“whirl sign”) and gallbladder distension are strong indicators for a GBV [3,14]. Strangulation of the cystic artery causing reduced vascularity of the gallbladder wall can be shown via Doppler ultrasound [7]. MRI may demonstrate twisting of the cystic duct and indicate the presence of necrosis while magnetic resonance cholangiopancreatography (MRCP) can show the relation of cystic duct to other components of the Calot triangle [1,2]. There is no role for endoscopic ultrasound in the diagnosis of GBV based on the current literature available. While laparoscopic detorsion and removal is recommended as it enables decompression to avoid bile duct injury, laparotomy may be performed in cases of massive distension [1,10].

Although the preoperative diagnosis of GBV has increased from 10% to 26% due to advancements in imaging modalities, GBV remains a difficult diagnosis owing to its similar presentation to acute cholecystitis [1,2]. Classical clinical features of GBV including patient symptoms and physical signs should prompt early suspicion of GBV for timely intervention to prevent life-threatening complications like gallbladder ischaemia, necrosis, perforation and biliary peritonitis [2,6,9].

## Conclusions

Clinical features of GBV which have been grouped into a "triad of triads" may be helpful; however, there is limited information regarding its sensitivity and specificity in the diagnosis of GBV. Further studies are needed to help improve the diagnostic accuracy for GBV. This case highlights the difficulties in diagnosing GBV and the importance of considering GBV as a differential diagnosis in an elderly patient with a classic presentation of acute cholecystitis. While a broader range of differential diagnoses needs to be considered in the paediatric age group, the investigations and management approaches are similar, and urgent cholecystectomy is the treatment of choice for GBV for both adult and paediatric populations. Although key radiological and clinical features of GBV have been discussed in this article, the preoperative diagnosis of GBV remains challenging and more research will be beneficial to aid with the diagnosis of GBV in the future.
